# Items

**Published:** 1880-12

**Authors:** 


### Items.

THE TREATMENT OF WRITERS' PALSY, OR *Crampe des Eirwains*.

The symptoms of writers' palsy are well known and vary from the mild fatigue of the fingers employed in holding the pen in slightly developed forms of the trouble, to severe pain or ache of the overused digits, with tender points over several articulations and occasional ache of the entire extremity. There is rarely a true paralysis, but, in aggravated cases, mild arthritis, usually of the right first carpo-metacarpal or metacarpo-phalangeal articulation, due without doubt to the fact that the pen or pencil, when supported in the ordinary Spencerian position as taught in the schools, while guided by the extremeties of the three first fingers, requires that the pressure of the second and third should be completely antagonized by the first or the thumb. Morcover the action of the hand in chirography is invariably from left to right of the page and it thus becomes necessary for the thumb to do the exclusive work of pushing the pen the entire distance, as the other fingers are, in consequence of their position, incapable of exciting any tractile face—that is when the fingers alone perform the task. When the motions of the pen are performed by the muscles of the arm or forearm the strain of course falls upon the latter.

The relief of this condition, never accomplished while the exciting cause is in operation, calls for rest of the affected part; and this demand, when the writer is supporting himself, or as is often the case, others besides himself, by his penmanship, is frequently uttered in vain. The prompt and permanent relief of the entire trouble by the use of the type-writer is now an acknowledged fact.

It is proper to add that this statement is made, not for the purpose of enumerating the merits of this exceedingly ingenious piece of mechanism, but to call attention to its remarkable value to those afected with writers' cramp. We are not in communication with those engaged in the sale of the instrument nor are these paragraphs penned under the stimulus of any of those ingenious promptings which the modern advertiser has made familiar to the public. The opinion expressed here is based upon a knowledge of some fifteen cases, in each of which the writer was surprised and delighted with the relief attained.

It is well known that the keys of the type-writer are moved by both hands and the work of each may be divided between as many fingers as the skill of the operator can employ. The labor is then distributed to both superior muscles, and, what is more, the gentle force required is not lateral, as in penmanship, but in the direction of gravity. Thus it happens that the work of an entire business day is accomplished with less fatigue than an hour's labor with the now old-fashioned pen.

